# Proteomics Reveal the Profiles of Color Change in *Brunfelsia acuminata* Flowers

**DOI:** 10.3390/ijms20082000

**Published:** 2019-04-23

**Authors:** Min Li, Yueting Sun, Xiaocao Lu, Biswojit Debnath, Sangeeta Mitra, Dongliang Qiu

**Affiliations:** College of horticulture, Fujian Agriculture and Forestry University, Fuzhou 350002, China; liminzyl@sina.com (M.L.); yuetingsun@126.com (Y.S.); xc531599541@126.com (X.L.); biswo26765@yahoo.com (B.D.); sangeeta.dae@hotmail.com (S.M.)

**Keywords:** *B. acuminata* petals, MALDI-TOF/TOF, GC-TOF-MS, qRT-PCR, differential proteins

## Abstract

*Brunfelsia acuminata* is a popular ornamental plant with different colors resulted from the rapid change of color after blooming. The petals at day one (purple), day three (white and purple) and day five (white) were used to analyze the reason of flower color change by a comparative proteomics approach, gas chromatography coupled to a time-of-flight mass analyzer (GC-TOF-MS) and quantitative real-time PCR (qRT-PCR). The results showed that the 52 identified proteins were classified into eight functional groups, 6% of which were related to the anthocyanin metabolic pathway. The expression levels of all anthocyanin proteins from the first day to fifth day were remarkably down-regulated, which was consistent with the changing patterns of the key genes (*CHS*, *CHI* and *F3′5′H*) in petals. Simultaneously, the main floral volatile components including Linalool and 2-Hexenal (E) were identified, and the contents of 2-Hexenal at day five increased dramatically. Moreover, the content of flavonoids and total phenolic increased at day five. The majority of the proteins associated with stress defense and senescence proteins were up-regulated and the activities of peroxidase (POD), superoxide dismutase (SOD) and catalase (CAT) in the petals at day five were significantly higher than others. It was concluded that the competition in the precursors of metabolic pathways occurs and causes the flow of metabolite to the pathways of floral scent and lignin derived from the shikimate pathway or degrade into others. Therefore, the anthocyanin content significantly decreased, and the petal color changed from deep purple to white.

## 1. Introduction

*B. acuminata* is an evergreen shrub native to Brazil. The date of flower blooming is from April to May (South of China). The color change in plants is very obvious, with a high ornamental value [1]. The color of the petal begins to change gradually from purple to white after two to three days of opening. The flower color is affected by different kinds of pigments, and the change is related to the decline of the anthocyanin content [2]. In general, the pigment of the petals is distributed in the vacuoles of epidermal cells, but is also present in other tissues, such as the cell wall [3], palisade, and chromoplasts [4]. The anthocyanin component in the *B. acuminata* petal includes in malvidin-3-*O*-glucoside chloride, petunidin-3-glucoside and delphinidin-3- glucoside, which are part of the polyphenolic [5]. They are water-soluble plant pigments that are susceptible to change [4] by pollinators [6], temperature, light [7], solvents, chemical structures, and pH changes [8]. Phenolic compounds are a large class of plant secondary metabolites including phenolic acids, tannins, lignans, coumarins, and flavonoids, which are responsible for the color of fruits and substrates for enzymatic browning [9]. Previous studies have shown that phenolic compounds play a role in the antioxidant activity of the flower [10,11]. Flavonoids are a biologically important group of phenolics in plants [12]. Secondary compounds are important in plants, especially in anthocyanin and as a stress and defense substance [13]. In addition, in the process of flower growth and development, the appearance of the petal changes with the alternation of the internal structure. Many studies have observed change in the structure and ultrastructure of the petals [14,15].

The biosynthetic pathway of anthocyanin has been the subject of much research and the associated biosynthetic and regulatory genes such as chalcone synthase (*CHS*), chalcone isomerase (*CHI*), and flavonoid 3′5′-hydroxylase (*F3′5′H*) are well defined in *Brunfelsia* plants [1,16,17]. These genes are also studied in many plants such as *P. hybrida ‘Mirage Rose’* [18], *Lilium* spp. cultivar ‘Dizzy’ [19]. The process of anthocyanin degradation in *B.calycina* was dependent on de novo synthesis of mRNAs and proteins of peroxidase (BcPrx01) [20]. It is speculated that POD is an enzyme that causes the degradation of anthocyanin of *B. calycina* petals by Oren-Shamir [2].

Proteome approaches are a powerful tool and can assist the investigation of comprehensive protein expression profiles in specific biological responses [21]. Two dimensional electrophoresis (2-DE) is one of the mainstream methods for floral proteomics, and has been widely applied to flower organs, such as the androecium, the gynoecium, and petals [22].

However, the detailed knowledge of the degradation of anthocyanin is poorly understood in the *B. acuminata* petal. Therefore, the aim of this study was to explore the profiles of color changes of the flower in *B. acuminata* through proteomics analysis.

## 2. Results

### 2.1. Changes in Corolla Diameter, Content of Water, Anthocyanin, Flavonoid, Total Phenolic, and Ultra-Structure during Flower Development

**Figure 1:**
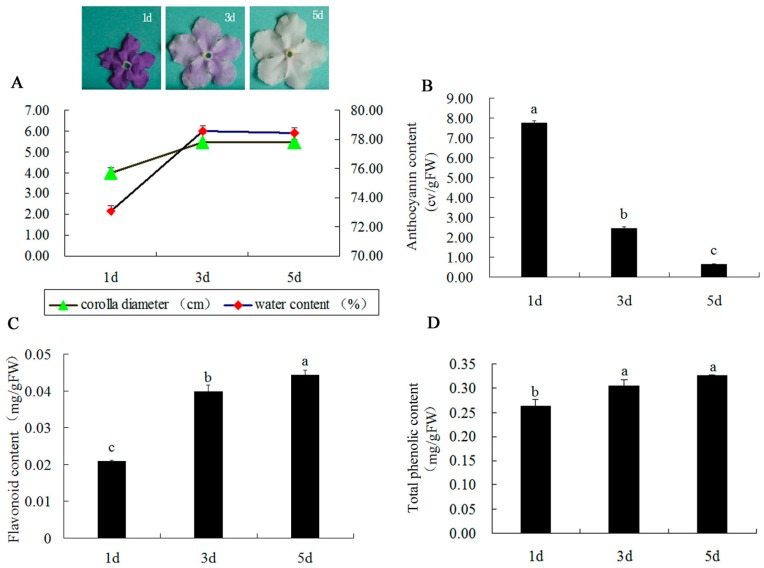
Changes of corolla diameter, water content, anthocyanin content, flavonoid content, and total phenolic content of *B. acuminata* petals. The different petals during anthocyanin degradation and the corolla diameter and water content changes after flower opening in petals (**A**); the contents of total anthocyanin (**B**), flavonoid content (**C**), and total phenolic content (**D**) in the petals. Note: Values (mean ± SD) were determined from three independent experiments (*n* = 3). Different letters above the bars indicate a significant difference at *p* < 0.05.

The corolla diameter of *B. acuminata* petals increased, and its water content at day 3 went up significantly as compared with day 1 and it kept stable in the petals at day 5 (Figure 1A). Simultaneously, the anthocyanin content reduced significantly (Figure 1B) in development of *B. acuminata* petals. The content accumulated to a maximum level at day 1 and degraded to a minimum level at day 5, whereas, the flavonoid content in the petals increased significantly from day 1 to day 3 (Figure 1C). Total phenolic content in the petals at day 3 was significantly higher than that in day 1. No significant difference was observed in between the contents at day 3 and at day 5 (Figure 1D).

**Figure 2:**
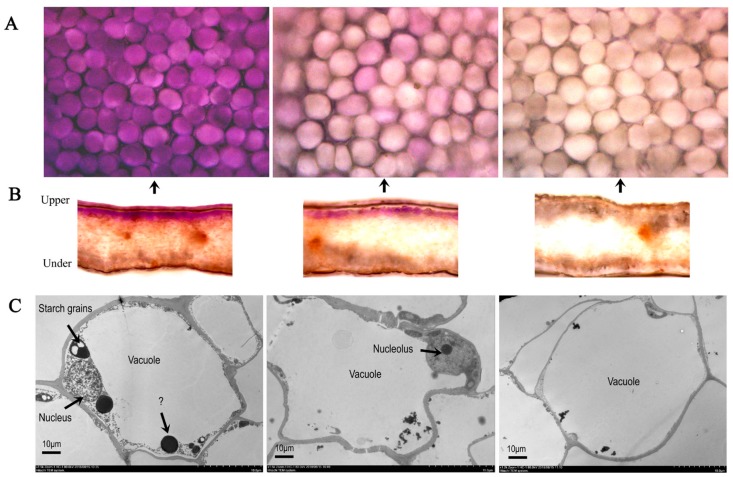
The pigment distribution and ultrastructure of epidermal cells in different days. Longitudinal section of the petal epidermis (**A**) and the petal upper epidermis under a microscope magnified 20 times (**B**); the petals of ultrastructure in upper epidermis of 10 µm (**C**).

From the day 1 to day 5, the cell volume of the petal expanded, and the petals grew rapidly (Figure 2A). The epidermal cell at day 1 was small purple and compact. With the expansion of the cell, its color became shallow at day 3, and became white at day 5 (Figure 2A,B). Unknown black pigment grain in the vacuoles of the petals was obviously observed at day 1, but the grain became smaller at day 3 and disappeared at day 5 (Figure 2C).

### 2.2. Protein Identification and Functional Classification

As shown in Supplementary Materials, 60 spots of the differentially abundant proteins were screened out. Figure 1 and 52 proteins were identified in *B. acuminata* petals by matrix-assisted laser desorption/ionization time-of-flight mass spectrometry (MALDI-TOF/TOF-MS) (Table 1). Among of which, 35 protein spots were significantly up-regulated and 17 protein spots were down-regulated. Of the 52 proteins successfully identified, some were identified as the same protein such as adenosine succinate syntheses (spot 3, spot 4), mitochondrial ATP synthase beta subunit (spot 32, spot 45), and anthocyanin 5-*O*-glucosyltransferase (5-GT, spot 17, spot 18). We found that 35 proteins were significantly up-regulated at day 5. Many of them had 2-fold or more in abundance. The six proteins (spot 25, spot 40, spot 46, spot 53, spot 27, spot 47) appeared at day 3 (Figure S2). Among of which, three new proteins (spot 27, spot 46, spot 47) showed a sudden increase in expression at day 3.

**Figure 3:**
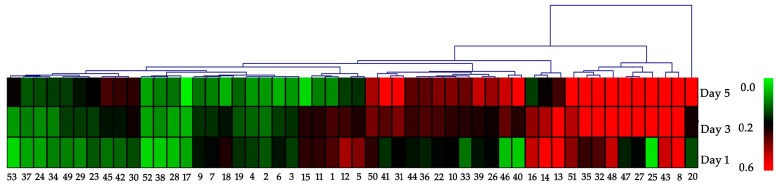
The heat map visualization of differential abundance of the identified 52 proteins in *B. acuminata* petals. The upregulated and downregulated proteins are indicated from red to green, respectively. The color scale is shown at the left of the cluster.

**Figure 4:**
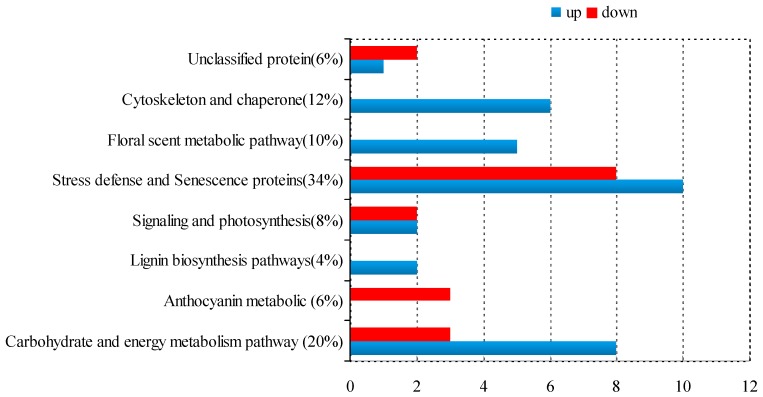
Functional classification and expression of identified proteins in *B. acuminata* petals.

The visualization of differential abundance of the identified 52 proteins was showed in Figure 3. It can be classified into eight groups: Carbohydrate and energy metabolism pathway (20%), anthocyanin metabolic pathway (6%), lignin biosynthesis pathway (4%), stress defense and senescence proteins (34%), floral scent metabolic pathway (10%), signaling and photosynthesis (8%), cytoskeleton and chaperone (12%), and unclassified protein (6%) (Figure 4). It is interesting to find that all the proteins involved in floral scent metabolic pathway, lignin biosynthesis pathways and cytoskeleton and chaperon were up-regulated, while all anthocyanin metabolic proteins were down-regulated (Figure 4).

### 2.3. The Expression Levels of Key Genes Encoding Anthocyanin Synthesis in Different Days

**Figure 5:**
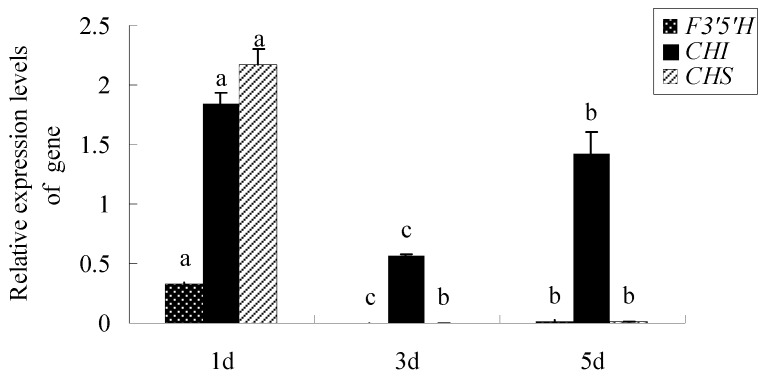
The expression levels of key genes of anthocyanin synthesis in different days. Values (mean ± SD) were determined from three independent experiments (*n* = 3). Different letters above the bars indicate a significant difference at *p* < 0.05.

The key genes of anthocyanins biosynthesis genes encoding proteins, namely, chalcone isomerase (*CHI*), flavonoid 3′5′-hydroxylase (*F3′5′H*), and *CHS* were examined to ascertain whether the protein differential abundance levels correlated with their mRNA content. Figure 5 showed that the expression levels of *CHS* and *F3′5′H* were remarkably down-regulated in different days. These results were consistent with trends in changes in color-related proteins. *CHI* has an upward trend in the later stage at day 5, which is likely to participate in other metabolic pathways.

### 2.4. Analysis of Volatiles

**Figure 6:**
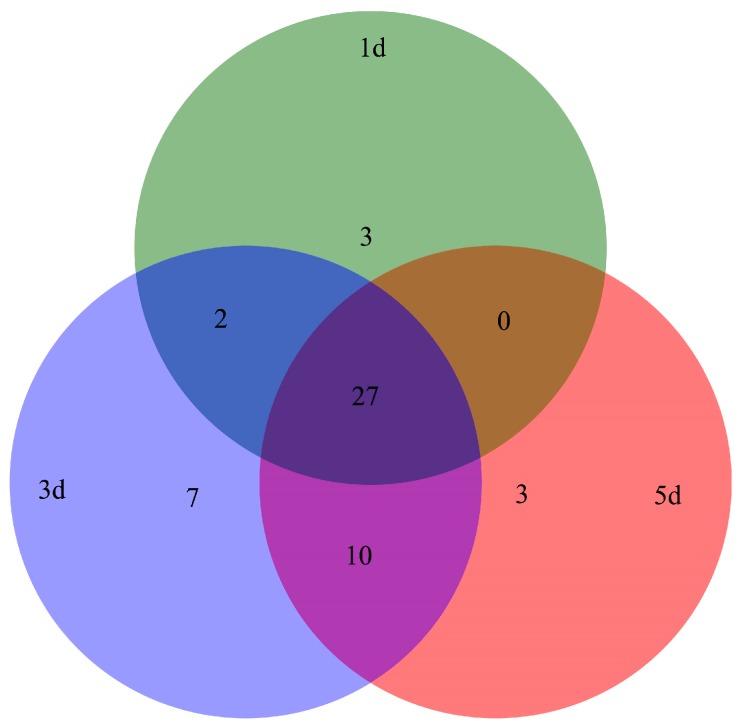
Volatile components were detected in different days of *B. acuminata petals*.

A total of 52 kinds of volatile components detected in different days of *B. acuminata* by GC-TOF-MS were classified into included terpenes, alcohols, aldehydes, esters, and so forth. At day 1, day 3, and day 5, 32, 46, and 40 components were detected in petals, respectively (Figure 6, Table S2). In Table 2, eight kinds of terpenoid with similarity greater than 800 and relative content greater than 5% is listed, and other volatiles are shown in Table S2. The volatile components of petals at day 1 are mainly linalool, and benzaldehyde and at day 3 are mainly including linalool, 2-hexenal, (E)-, trans-Linalool oxide (furanoid), where relative content of the compound is more than 10%. The relatively high content of the components at day 5 was linalool, 1-hexanol, benzeneacetaldehyde, which were 19.24%, 12.53%, and 11.40%, respectively.

### 2.5. SOD, CAT, POD Activity and Soluble Protein Content in Petals of B. acuminata

**Figure 7:**
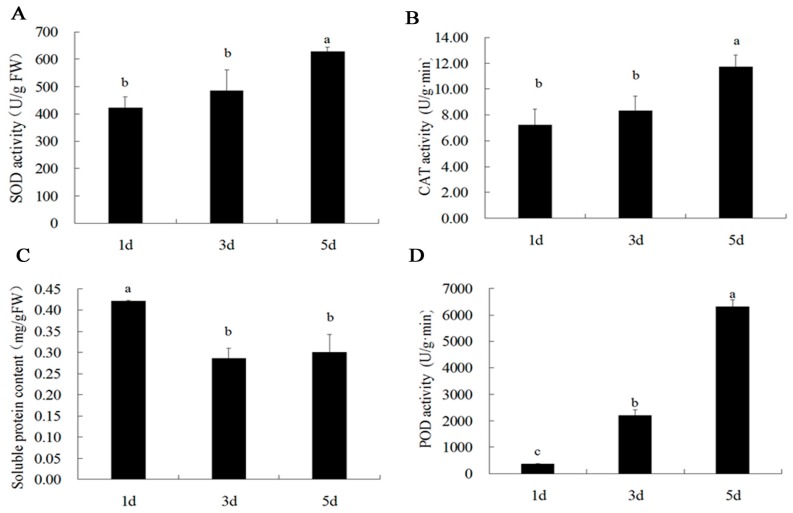
Activity of antioxidant-related enzymes, superoxide dismutase (SOD) (**A**), catalase (CAT) (**B**), soluble protein content (**C**), peroxidase (POD) (**D**), and in *B. acuminata* petals during blooming. Values (mean ± SD) were determined from three independent experiments (*n* = 3). Different letters above the bars indicate a significant difference at *p* < 0.05.

Superoxide dismutase (SOD) and catalase (CAT) activity in the petals at day 5 were significantly higher than those at day 1 (Figure 7A,B). However, the soluble protein content (Figure 7C) in the petals decreased significantly from day 1 to day 5. The content had no significant difference between day 3 and day 5. Interestingly, the peroxidase (POD) activity increased significantly from day 1 to day 5 during blooming (Figure 7D).

## 3. Discussion

### 3.1. Main Proteins and Genes Related to Anthocyanin Synthesis

The appearance of plant color is closely related to the content of anthocyanin. In most cases, the color change is due to the induction of anthocyanin synthesis, but the color change of *B. acuminata* petal during anthesis is the exact opposite of *Viola cornuta cv.* yesterday, today, and tomorrow [23]. In the process of *B. acuminata* flowering, anthocyanin biosynthesis in the early opening petal of *B. acuminata* is the most exuberant, resulting in the deepest color. The anthocyanin degraded gradually, resulting in a change of flower color from deep purple to white. The color change of the petal is a complex process, from flower formation to degradation, requiring the participation of many enzymes and genes.

The enzymatic degradation of anthocyanin in plant tissues can play an important role in the regulation of plant pigments. Two proteins namely, anthocyanin-5-*O*-glucosyltransferase (5-GT) and anthocyanin-O-methyl transferase (OMT) related to the metabolic pathway of anthocyanin were identified and analyzed in three different periods of discoloration of *B. acuminata* petals (Table 1, Figure 3). 5-GT (spot 17, spot 18) is an enzyme that forms anthocyanin-3,5-O-diglucoside from anthocyanin-3-O-glucoside, which is responsible for the modification of anthocyanins to more stable molecules in complexes for co-pigmentation, supposedly resulting in a purple hue [24]. In our study, the expression level of 5-GT was higher in purple petals (day 1, 1d) than that in white petals (day 5, 5d) (Figure 3). OMT (spot 14) is one of the key enzymes for anthocyanin modification and flower pigmentation [25]. Many OMT genes are involved in the formation of methylated anthocyanins [25]. OMT was down-regulated and may not be able to methylate to form anthocyanins. Furthermore, all anthocyanin metabolic proteins were down-regulated, and many anthocyanin-modifying enzymes involved in the anthocyanin synthesis pathway were not activated during the petals changing from purple to white.

*CHS*, *CHI*, and *F3′5′H* are important enzymes for the formation of flower colors [26]. F3′5′H is a key enzyme for the synthesis of blue-purple pigments [27]. The expression levels of *CHS* and *F3′5′H* are both down-regulated in the process of flowering, consistent with protein differential abundance trends (Figure 5, Table 1). This result was supported by our previous work that the striking color change from dark purple to pure white resulted from a decline in anthocyanin content of the petals and was preceded by a decrease in the expression of *BaCHS* [28]. However, *CHI* expression differed from them, which has an upward trend in the later stage at day 5. Chalcone isomerase (CHI) converts yellow chalcone to colorless naringenin, which expression level directly affects the accumulation of yellow chalcone, a colorless phenotype, and flavanol compounds [29,30]. Maybe *CHI* is involved in other metabolic pathways, and the expression level will decrease later.

### 3.2. Other Protein Associated with Anthocyanin Synthesis

**Figure 8:**
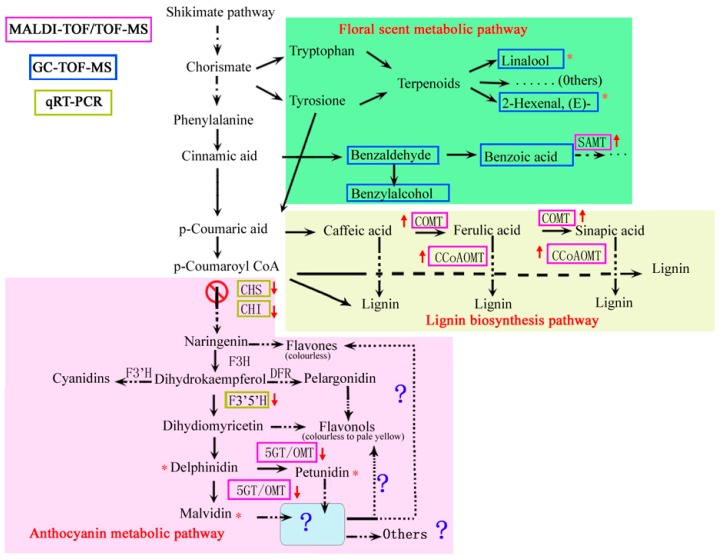
A putative metabolic map described the pathway including the floral scent metabolic pathway and lignin pathway during the degradation of anthocyanins in *B. acuminata* petals. The diagram shows the different pathways. The diagram summarizes the results of qRT-PCR, MALDI-TOF/TOF and GC-TOF-MS analyses. The dashed black arrows represent several consecutive enzymatic steps. The color of the box indicates the method by which they were identified. The red arrow represents up or down and the symbol with * in the picture is expressed as an important substance identified in the petals. Enzymes: SAMT, salicylic acid carboxyl methyltransferase; COMT, catechol O-methyl transferase; CCoA-OMT, caffeoyl-CoA O-methyl; OMT, Anthocyanin-*O*-methyl transferase. Genes: *CHS*, chalcone synthase; *CHI*, chalcone isomerase; *F3′5′H*, flavonoid 3′5′-hydroxylase.

The shikimate pathway is induced at later stages in the flower of *B. acuminata*, linking carbohydrate metabolism to the precursors for the synthesis of anthocyanins, benzenoids, and lignin [31,32] (Figure 8). As a class of floral substances in plants, terpenoids have similar synthetic pathway with anthocyanins and carotenoids [33]. In our study, we found that four proteins are terpenoid-related and up-regulated during the flowering. Methyl salicylate is a volatile plant component, but also an important substance in the defense mechanism of plants [34]. The methylation of salicylic acid is performed by salicylate carboxymethyltransferase (SAMT, spot 39) [34]. 1-hydroxy-2-methyl-2-(E)-butenyl 4-diphosphate reductase (IDS, spot 33), and 1-deoxy-D-xylulose-5-phosphate reductoisomerase (DXR, spot 34) were significantly up-regulated, indicating that terpenoids continue to increase from day 1 to day 5. The putative salicylic acid carboxyl methyltransferase (spot 40, spot 42) is a new protein related to floral scent and lignin pathway and is also up-regulated. In the process of petal scent release, the black pigmentation is reduced and finally disintegrated (Figure 2). It can be speculated that the function of the black pigmentations is a reservoir of aroma precursors or energy in *B. acuminata*. This black pigmentation is similar to the description of the epidermal cells of jasmine petals described by Zhang [35]. In the *B. acuminata* petals, the expression levels of caffeoyl O-methyltransferase (COMT, spot 41) and caffeoyl-CoA O-methyltransferase (CCoAOMT, spot 47) were significantly up-regulated. Both are involved in the synthesis of the volatile compounds in *Brunfelsia* [31]. A similar result has been reported that multifunctional CCoAOMTs play roles in catalyzing the 3′or 3′-5′ O-methylation of their B ring of flavonoid substrates [36]. Glutathione S-transferase is associated with the formation of color, which can transport anthocyanins into vacuoles. 14-3-3-like protein GF14 Psi (spot 22) is associated with the shikimic acid pathway, which participates in the synthesis of aromatic compounds and indirectly affects the color changes [37]. GST and Glutamine synthetase 1, 4 (GS1, 4, spot 27) showed a significant upward trend in *B. acuminata* petals.

Lignin biosynthesis is the second metabolic pathway of the phenylpropane pathway branch, which is induced during petal expansion in the opening *Brunfelsia* flower [31]. Both anthocyanins and benzoic acid are derived from benzoic acid metabolic pathways, and there is a competitive effect between them [38]. Inhibition of other competitive biosynthetic pathways in the synthesis of floral substances can improve the synthesis of plant floral compounds such as anthocyanin biosynthetic pathway [38]. In our experiment, lignin synthesis enzymes and floral synthesis-related proteins were up-regulated, while anthocyanin synthesis-related proteins showed down-regulation, which may be the key reason to color changes. The dramatic changes in color are accompanied by the synthesis of aromatic phenol volatiles in the petal of *B. acuminata*. Anthocyanin degradation and the synthesis of aromatic benzene compounds may be pollinators’ signals [1,31]. Further studies may reveal a network of *B. acuminata* and related species of volatile and pigment phenolic metabolism.

The anthocyanin biosynthetic pathway belongs to a branch of the flavonoid biosynthetic pathway. Based on flavanones, all other flavonoid-classes are generated, including isoflavones, flavanols, anthocyanidins, flavanols, and flavones [39]. The anthocyanin content dropped significantly (Figure 1B), while the contents of flavonoid and total phenolic in the petals of *B. acuminata* increased significantly from day 1 to day 3 (Figure 1C). Thus, we speculated that the competition in the precursors of metabolic pathways occurs and causes the metabolite flow to the pathways of floral scent and lignin both derived from the shikimate pathway (Figure 8).

### 3.3. The Proteins Associated with Other Metabolic Pathways

In plants, there are many enzymes involved in sucrose metabolism, among which invertase is one of the key enzymes involved in plant sucrose metabolism [40]. Vacuolar invertase 2 (spot 29) can be the one in vacuole hydrolyzing glucose and fructose to regulate the concentration of intracellular sucrose. In this study, the water content of *B. acuminata* petals at day 3 went up significantly and it kept stable in the petals at day 5 (Figure 1A,B), and the vacuolar invertase was up-regulated from day 1 to day 5, promoting the expansion and enlargement of the petals.

Soluble acid invertase 2 (spot 24) is also a key enzyme in the process of carbohydrate metabolism, mainly in the vacuole. Highly active soluble acid invertase is closely related to the growth of the young parts of the plant and the rapid expansion of the storage organs [41].

With the growth of *B. acuminata* petals, respiration will become faster to provide the energy and raw materials necessary for plant life activities. Malate dehydrogenase, cytoplasmic-like (MDH, spot 5) is present. In this study, the protein differential abundance of MDH decreased from purple to white in the petals (Figure 3). It is possible that the petals are about to enter the aging period and the petal respiration and energy metabolism begin to weaken on day 5 of bloom. The ATP synthase β subunit (spot 49) was up-regulated in the *B. acuminata* petals, which indirectly provided energy for petal growth and development.

It is worth noting that Phosphoenolpyruvate carboxylase kinase1 (PpcK1, spot 46) and Glutamine synthetase (GS, spot 27) showed a sudden increase in expression at day 3. Two novel proteins, ASR1 (spot 25) and plastid-lipid-associated (spot 53) are related to stress defense proteins, which also appeared and up-regulated at day 3 (Figure S2). Phosphoenolpyruvate carboxykinase (PEPCK) is only known to be located in the cytosol in flowering plants [42]. In CAM plants, it is an important metabolic regulated and Ca^2+^-independent protein related to light, and the shift of kinase phosphorylation signal transduction pathway [43]. GS is a crucial enzyme in the network of N metabolism and also involved in N recycling in the plant [44]. ASR1 belongs to a family of hydrophilic proteins in responses to abiotic stresses as well as signaling molecules [45]. Plastid lipid-associated proteins are known to accumulate in fibrillar-type chromoplasts such as in leaf chloroplasts from *Solanaceae* plants under abiotic stress conditions [46] and involved in the pigment accumulation during fruit development [47]. The majority of these proteins associated with stress defense and senescence proteins were up-regulated indicating that the petal of *B. acuminata* at day 5 might be at the beginning of aging (Figure 4). This hypothesis is supported by the data that the activities of POD, SOD, and CAT in the petals at day 5 were significantly higher than those at day 3 and day 1 to prevent ageing (Figure 7) [48,49], and that all the protein involved in cytoskeleton and chaperon were up-regulated (Figure 4). The cytoskeleton constitutes the structural support of the living matter. Molecular chaperones are housekeeping factors of the cytoskeleton network [50].

In conclusion, the color change of *B. acuminata* flower is a complicated process involving numerous factors. The competition in the precursors of metabolic pathways occurs and causes the metabolite flow to the pathways of floral scent and lignin both derived from the shikimate pathway. Meanwhile, the decline of expression levels of *CHS*, *CHI*, and *F3′5′H* resulted in down-regulation of anthocyanin metabolic proteins. Therefore, the anthocyanin content decreased, and petal color change from deep purple to white (Figure 1). This work provides new proteomic and GC-TOF-MS insights of color change of flower in plants.

## 4. Materials and Methods

### 4.1. Plant Materials and Morphological Indicators

Flowers opened with purple petals (day 1,1d) that changed to light purple and white (day 3,3d), and pure white (day 5,5d) during a 5-day lifespan. *B. acuminata* petals in the different stages (1d, 3d, 5d) were collected from the greenhouse of Fujian Agriculture and Forestry University, Fuzhou, China (Figure 1A). The plants were grown at 20–25 °C/12–15 °C (day/night) temperature conditions, and 60%–80% humidity, and 14/10 h (day/night) with 100 mol·m^−2^·s^−1^ photosynthetically active radiation (PAR). Three replicate samples were collected at random from individual plants and immediately kept in liquid nitrogen, and stored at −80 °C for proteomic, quantitative real-time (qRT-PCR), and GC-TOF-MS analysis.

The petals of *B. acuminata* were randomly selected to observe their morphological changes and measure their flower diameter and water content. Vernier caliper was used to measure corolla diameter. Petal water content was determined as the percentage of total petal weight ((FW-DW)/FW·100) by weighing samples of 5 outer flower petals, before and after their drying in a drying box at 60 °C for 20 min. Each measurement was repeated with 5 flowers [51].

### 4.2. Observation of Petal Epidermal Cells Structure and Ultra-Structure

The petals were washed with distilled water. The longitudinal section of the petal was made by double-blade cutting to determine the internal structure of the petal and the distribution of pigment. A temporary water-filled sheet was prepared to observe the shape of the epidermis cells of the petals and whether the epidermis had a pigment distribution by optical microscope (Nikon, Tokyo, Japan). The photographs were taken under a microscope magnified 20 times. Transmission electron microscope (TEM, Hitachi, Tokyo, Japan) was used to observe the changes of epidermal cell ultra-structure of a flower in different days. The middle part of the fresh flowers were cut into pieces (about 1 mm^2^), and these fragments of flower were used for transmission electron microscopy in accordance to the described method by Meng [52].

### 4.3. Preparation of Total Protein Extraction

Proteins samples were prepared by phenol extraction in accordance with the methods of Wang [53]. The protein sample was dried at −20 °C and stored at −80 °C until used.

### 4.4. 2-DE and MALDI-TOF/TOF Analysis

The protein powder was mixed with a lysis buffer, containing 7 M urea, 2 M thiourea, and 4% CHAPS, 2% Pharmalyte3-10, and 40 mM DTT, and ultrasonically shaken for 20 min. After the protein powder was completely dissolved, it was placed in a water bath at 37 °C for 2.5 h, centrifuged at 20,000× *g* for 15 min at room temperature. The supernatant was transferred to a new centrifuge tube after centrifugation. The protein content was determined according to the Bradford method [54]. The steps of two-dimensional electrophoresis are in accordance with Wang et al [53]. A volume of 1.5 mg aliquots of protein was added to a 24 cm linear gradient Immobilized *p*H gradient (IPG) strip at pH 4–7 and hydrated for 12 h. The isoelectric focusing procedure: 200 V (l h)–500 V (l h)–1000 V (l h) -Gradient–8000 V (0.5 h)–8000 V (6 h)–1000 V (2 h). The maximum current for each strip is 50 mA. The operating conditions were 20 °C.

After the first isoelectric focusing, the strips were equilibrated in equilibrium solution I (50 mM Tris-HCl (pH 8.8), 6 M urea, 30% *v*/*v* glycerol, 2% *w*/*v* SDS, 1% DTT) for 15 min and transferred to equilibrium solution II (50 mM Tris-HCl (pH 8.8), 6 M urea, 30% *v*/*v* glycerol, 2% *w*/*v* SDS, 2.5% iodoacetamide) for 15 min. After the end of the two balances, the strips were removed, and the surface of the strip was washed again with electrophoresis buffer and transferred to a 12% sodium dodecyl sulfate polyacrylamide gel electrophoresis (SDS-PAGE) in Ettan DALT-six System (GE Healthcare Bio-Sciences, Uppsala, Sweden) at 18 °C.

The electrophoresis was terminated when the bromophenol blue band reached the bottom of the gel. After electrophoresis, staining was performed by Coomassie brilliant blue staining (CBB-R250) and shaking for 2 h. After staining, the stained liquid drained, the gels washed with distilled water and then added the right amount of bleaching solution for decolorization, until the gel background cleared up. After the decolorization was completed, the Epson scanner 11000XL-USB (EPSON, Tokyo, Japan) was used to perform gel image scanning. Gel image analysis was performed using Image Master^TM^ 2D Platinum Software Version 5.0 software (BioRad, Hercules, CA, USA).

The differential protein spots were excised carefully from the gels from the preparative 2D gels. In-gel digestion by trypsin and analysis by MS analysis in an AB SCIEX MALDI TOF-TOF™ 5800 analyzer (AB SCIEX, Foster City, CA, USA), was performed in accordance with the method described by Wang [53]. According to the results of mass spectrometry analysis, the data were selected using GPS Explorer (V3.6, Applied Biosystems) and the search engine MASCOT (2.1, MatrixScience, London, UK) with the following parameters: National Center for Biotechnology Information green plants (release date: 2015. 04. 01); MS tolerance was set at 100 ppm; MS/MS tolerance of 0.8 Da. Proteins were considered statistically significant (*p* < 0.05 and Ratio > 1.5) confident identifications with scores greater than 75. Individual raw MS and MS/MS spectra were accepted if at least two identified peptides having a probability of 95% correct match were found.

### 4.5. GC-TOF-MS Analysis

The flower volatile compounds were carried out on an Agilent 7890 gas chromatography (Agilent, Santa Clara, CA, USA) unit and Gerstel Autosampler (Gerstel, Muhlheim, Germany), and a LECO Pegasus HT time of flight mass spectrometer (LECO, St. Joseph, MI, USA) as described by Barakiva [31]. Individual flowers collected from day 1 to day 5 were placed in a 10 mL glass, sealed, and incubated under ambient conditions.

The identification of individual compounds was detected and identified by comparison with the mass spectra library from National Institute of Standards and Technology (NIST, Gaithersburg, MD, USA) and Fiehn Retention Time Lock (Agilent, Santa Clara, CA, USA) library. All the available data acquisition and processing were conducted using Chroma TOF-GC software version 4.51.6.0 (Leco, MI, USA) and performed ANOVA statistical analysis for significance and principal component analysis with a similarity greater than 800.

### 4.6. Quantitative Real-Time (qRT-PCR) Analysis

The qRT-PCR analysis in accordance with the method described by Li et al. [28] The *CHS*, *CHI*, and *F3′5′H* genes sequences for primer design were obtained from GenBank (Genbank accession number: JN966986, JN887637, JQ678765), respectively. All of the primers used are shown in Table S1. Relative transcription levels were calculated using the 2^−ΔCTΔCT^ method.

### 4.7. Analysis of Physiological Parameters

Total contents of anthocyanin and flavonoid were determined according to the method of Zhang [55] with slight modifications. In brief, 0.1 g of each tissue was ground in liquid nitrogen and total anthocyanins were extracted with HCl/methanol (1:99, *v*/*v*) at dark for 4 h. The supernatants were determined using UV spectrophotometry at 535 nm and 657 nm. Total phenolic content was determined according to Mitra [56]. Soluble protein content was determined by Coomassie brilliant blue staining method, SOD, POD and CAT activity was quantified according to Wang [57].

### 4.8. Bioinformatic Analysis of Identified Proteins

The clustering analysis and heat map of protein differential abundance were carried out using the expression values and generated with MultiExperiment Viewer version 4.8 software (MeV; http://www.tm4.org/mev/). The results were viewed using Java TreeView. The GO analysis and classification are based on KEGG (http://www.kegg.jp/kegg/pathway.html).

### 4.9. Statistical Analysis

All the indexes were analyzed and plotted by Excel 2003 (Microsoft Corporation, Seattle, WA, USA) and the difference was statistically analyzed by SPSS 19 software (IBM, Armonk, NY, USA). Duncan’s multiple range tests were considered statistically significant at *p* ≤ 0.05. The samples were measured from different plants, analyzed in triplicate (*n* = 3), and shown as mean ± SD.

## Figures and Tables

**Table 1:** Identification of proteins from *B. acuminata* petals using MALDI-TOF/TOF-MS.

Spot No ^a^	Protein Name	Species	Accession No.	MW (kDa)/*p*I ^b^	Score	Cov ^c^	Fold Changes ^e^	
**Carbohydrate and Energy Metabolism Pathway**								
D5 ^d^	malate dehydrogenase, cytoplasmic-like	*Solanum lycopersicum*	gi|460404529	35.361/5.91	235	25%	−2.33	−1.55
D10	2,3-bisphosphoglycerate-independent phosphoglycerate mutase-like	*Glycine max*	gi|356568270	60.799/5.58	247	11%	−1.92	−1.32
D19	3-isopropylmalate dehydrogenase, chloroplastic-like	*Solanum lycopersicum*	gi|460386440	43.396/6.05	232	14%	−2.06	−1.25
U23 ^d^	ATP synthase beta subunit	*Eleutherococcus senticosus*	gi|343410685	39.790/5.77	227	35%	1.20	1.18
U24	soluble acid invertase 2	*Orobanche ramosa*	gi|294612072	61.628/5.24	133	19%	2.07	1.51
U29	vacuolar invertase 2	*Gossypium hirsutum*	gi|268526570	69.303/5.14	185	8%	2.16	1.98
U32	ATP synthase subunit beta, mitochondrial-like	*Solanum lycopersicum*	gi|460382474	59.825/5.94	869	45%	2.26	1.46
U45	ATP synthase subunit beta, mitochondrial-like	*Solanum lycopersicum*	gi|460382474	59.825/5.94	311	26%	3.00	2.71
U46	phosphoenolpyruvate carboxylase kinase 1	*Clusia minor*	gi|39842451	28.716/6.38	88	22%	13.09	10.67
U49	ATP synthase beta subunit	*Eleutherococcus senticosus*	gi|343410685	39.790/5.77	227	29%	2.40	2.26
U52	1,2-beta-fructan 1F-fructosyltransferase	*Helianthus tuberosus*	gi|3367690	69.214/5.02	129	12%	2.11	2.00
**Anthocyanin Metabolic Pathway**								
D14	Anthocyanin-*O*-methyl transferase	*Solanum tuberosum*	gi|441433515	26.282/5.69	90	19%	−7.04	−1.04
D17	Anthocyanin-5-*O*-glucosyltransferase	*Petunia x hybrida*	gi|6683052	52.130/5.07	98	13%	−13.79	−1.04
D18	Anthocyanin-5-*O*-glucosyltransferase	*Petunia x hybrida*	gi|6683052	52.130/5.07	100	7%	−3.94	−1.36
**Lignin Biosynthesis Pathway**								
U41	caffeate-*O*-methyltransferase	*Liquidambar styraciflua*	gi|5732000	39.944/5.69	80	22%	4.70	3.77
U47	caffeoyl-CoA O-methyltransferase	*Broussonetia papyrifera*	gi|46394464	27.701/5.31	412	40%	5.25	3.44
**Stress Defense and Senescence Proteins**								
D1	polyphenol oxidase E, chloroplastic-like isoform 2	*Solanum lycopersicum*	gi|460401035	66.181/6.36	83	18%	−2.66	−1.02
D2	polyphenol oxidase	*Nicotiana tabacum*	gi|92919068	57.748/5.92	126	13%	−2.10	−1.02
D6	proteasome subunit alpha type-6-like	*Solanum lycopersicum*	gi|460412613	27.301/6.11	102	32%	−4.08	−1.37
U8	glutathione S-transferase	*Solanum commersonii*	gi|148616162	23.843/5.98	82	30%	1.34	1.53
D13	lactoylglutathione lyase-like	*Solanum lycopersicum*	gi|460373807	32.839/5.95	283	32%	−2.00	−1.07
U25	ASR1 protein	*Solanum ochranthum*	gi|321155417	12.547/6.48	307	34%	31.33	23.55
U28	CLPC	*Theobroma cacao*	gi|508775360	102.257/6.36	373	27%	3.45	1.12
U35	S-adenosyl methionine synthase-like	*Solanum tuberosum*	gi|78191442	43.189/5.52	466	40%	2.11	2.01
U43	glutathione S-transferase L3-like	*Cicer arietinum*	gi|502121795	27.092/5.79	91	15%	1.59	1.51
U44	putative glutathione S-transferase zeta-class 2	*Brassica napus*	gi|330250478	25.336/5.53	82	24%	2.14	1.49
D3	Adenylosuccinate synthetase, chloroplastic-like	*Solanum lycopersicum*	gi|460407669	55.408/7.55	87	14%	−2.39	−1.04
D4	Adenylosuccinate synthetase1, chloroplastic-like	*Solanum lycopersicum*	gi|460407669	55.408/7.55	93	18%	−2.11	−1.38
D15	Aspartic proteinase	*Theobroma cacao*	gi|508719874	54.428/5.56	55	3%	−7.85	−1.08
D16	endochitinase precursor	*Humulus lupulus*	gi|4960049	33.508/7.42	109	4%	−3.09	−1.10
U20	Small ubiquitin-related modifier 1	*Arabidopsis thaliana*	gi|21542462	10.969/4.91	77	48%	9.19	1.68
U26	annexin p34-like protein-like	*Solanum tuberosum*	gi|81074127	35.909/5.54	174	24%	2.01	1.59
U27	Glutamine synthetase 1,4	*Theobroma cacao*	gi|508707247	39.098/6.02	160	18%	5.21	2.86
U51	heat shock 70 protein	*Spinacia oleracea*	gi|2773050	76.094/5.19	554	25%	2.02	1.07
U53	Plastid-lipid-associated protein, chloroplast precursor, putative	*Ricinus communis*	gi|223536371	34.979/4.84	148	24%	2.06	1.78
**Floral scent Metabolic Pathway**								
U33	1-hydroxy-2-methyl-2-(E)-butenyl 4-diphosphate reductase	*Ipomoea batatas*	gi|325557690	51.682/5.90	163	27%	2.58	2.20
U34	1-deoxy-D-xylulose-5-phosphate reductoisomerase	*Solanum lycopersicum*	gi|350537527	51.465/5.94	266	27%	2.01	1.49
U39	SAMT	*Anthocercis littorea*	gi|58201456	32.353/4.79	151	22%	2.11	1.62
U40	Putative S-adenosyl-L-methionine:Salicylic acid carboxyl methyltransferase	*Pisum sativum*	gi|37725949	40.552/5.17	78	19%	15.05	7.00
U42	putative S-adenosyl-L-methionine:Salicylic acid carboxyl methyltransferase	*Pisum sativum*	gi|37725949	40.552/5.17	52	5%	2.23	1.56
**Signaling and Photosynthesis**								
D11	inositol-3-phosphate synthase	*Solanum lycopersicum*	gi|460388681	56.526/5.45	189	23%	−2.79	−1.02
D12	inositol-3-phosphate synthase	*Solanum lycopersicum*	gi|460388681	56.526/5.45	318	23%	−2.84	−1.51
U22	14-3-3-like protein GF14 Psi	*Eutrema salsugineum*	gi|309952059	28.752/4.78	166	48%	2.07	1.81
U31	ruBisCO large subunit-binding protein subunit beta, chloroplastic-like	*Cicer arietinum*	gi|502125499	62.800/5.85	348	22%	2.15	1.26
**Cytoskeleton and Chaperone**								
U30	chaperonin CPN60-2, mitochondrial-like	*Solanum lycopersicum*	gi|460404682	61.521/5.51	175	18%	2.37	1.99
U36	beta-actin	*Zoysia japonica*	gi|284157810	41.697/5.23	459	35%	2.21	2.11
U37	actin 6	*Populus trichocarpa*	gi|222860713	40.592/5.05	166	27%	1.85	1.43
U38	actin	*Gossypium hirsutum*	gi|32186904	41.878/5.39	82	27%	2.5	2.25
U50	60-kDa chaperonin-60 alpha-polypeptide precursor, partial	*Brassica napus*	gi|289365	57.657/4.84	403	24%	2.61	1.93
**Unclassified Protein**								
D7	predicted protein	*Physcomitrella patens subsp. Patens*	gi|162667966	28.780/5.38	79	11%	−2.13	−1.11
D9	putative transcription factor BTF3-like	*Solanum tuberosum*	gi|82623431	17.472/6.31	240	34%	−2.06	−1.12
U48	cp10-like proteinCP10	Gossypium hirsutum	gi|21780187	26.761/7.77	122	9%	1.30	1.73

^a^ Spot number corresponds to the 2-DE gel in Figure S1; ^b^ theoretical molecular mass (MW) and isoelectric point (pI) of the homologous protein calculated with a tool available at NCBInr database.; ^c^ sequence coverage; ^d^ D down-regulated proteins, U up-regulated proteins.; ^e^ ratio of protein levels compared to day 1 (left: Day 3/Day 1, right: Day 5/Day 1).

**Table 2:** Main terpenoids were detected in different days of *B. acuminata petals*.

Number	Compounds	Relative Contents (%)		
		1d	3d	5d
**1**	Linalool	37.59 ± 8.39	31.27 ± 2.12	19.24 ± 4.12
2	2-Hexenal, (E)-	44.92 ± 5.11	20.98 ± 1.29	—
3	trans-Linalool oxide (furanoid)	5.91 ± 1.47	11.61 ± 1.58	8.27 ± 1.56
4	(E)-4,8-Dimethylnona-1,3,7-triene	9.62 ± 5.28	9.81 ± 1.07	10.11 ± 6.31
5	2-Furanmethanol, 5-ethenyltetrahydro-à,à,5-trimethyl-, *cis*-	3.47 ± 0.42	9.34 ± 1.60	6.07 ± 1.37
6	1-Hexanol	5.22 ± 0.83	5.13 ± 1.25	12.53 ± 5.98
7	Benzeneacetaldehyde	0.67 ± 0.20	4.15 ± 1.70	11.40 ± 0.85
8	2-Hexenal	0.74 ± 0.44	0.45 ± 0.13	16.78 ± 1.43

## Supplementary Materials

Supplementary materials can be found at https://www.mdpi.com/1422-0067/20/8/2000/s1.
