# Supplementary material for: Proteomics Reveal the Profiles of Color Change in Brunfelsia acuminata Flowers
Source: Int J Mol Sci. 2019 Apr 23;20(8):2000. doi: 10.3390/ijms20082000 (PMC6514780; doi:10.3390/ijms20082000)
Supplement: Supplementary file 1 [file ijms-20-02000-s001.pdf]

Supplementary Material

1 Supplementary Figures and Tables

1.1. Supplementary Figures

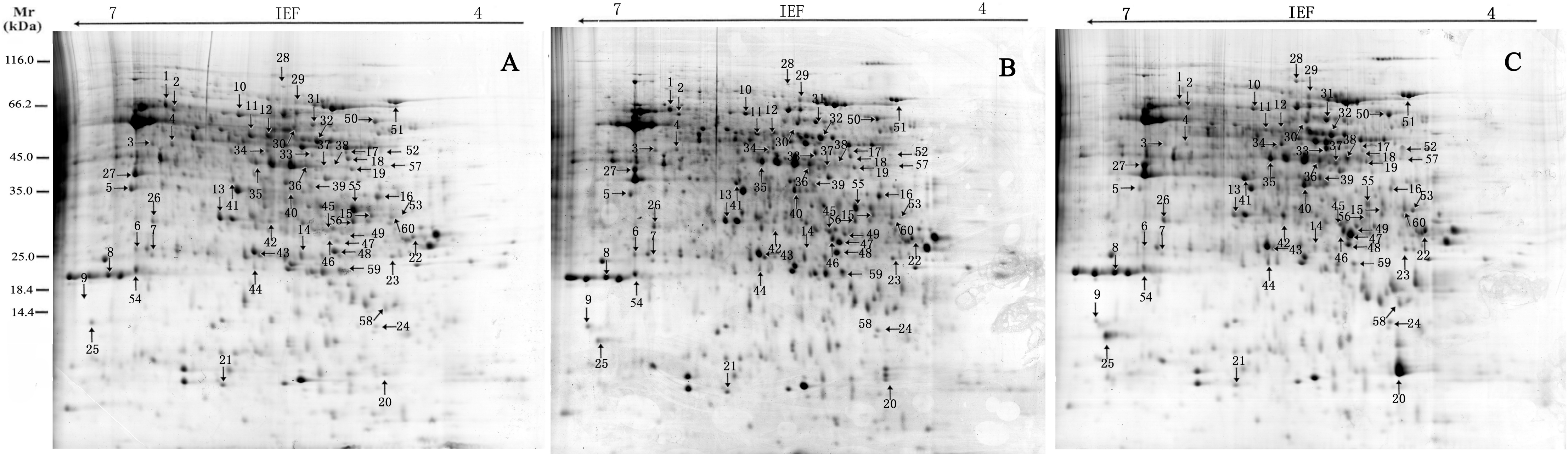

Supplenmentary Figure 1. 60 protein spots differentially expressed proteins were screened out and image analysis that changed 1.5 or more in abundance of B. acuminata. A: 1d; B: 3d; C: 5d

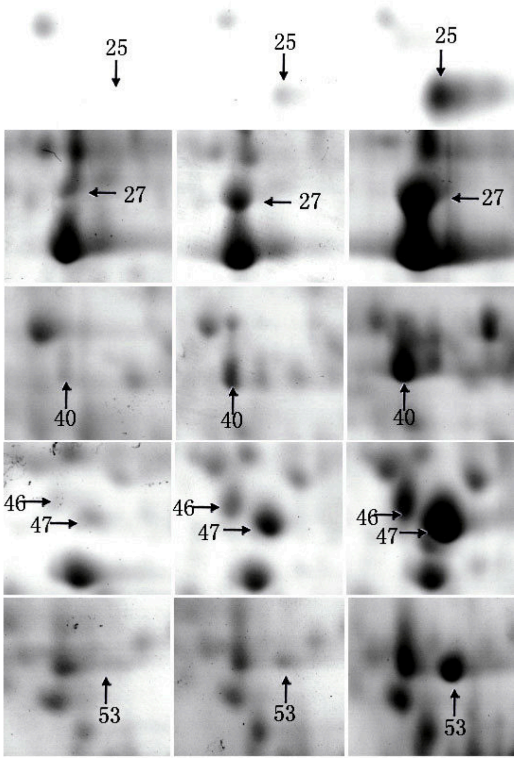

Supplenmentary Figure 2. The 6 proteins that stand out from day 1 to day 3 in the petals of *B. acuminata*

1.2. Supplementary Tables

Supplementary Table 1. Primer names and sequences.

Gene name	Primer Sequence (5'-3')
CHS-F	CAAAGGAGGGCTTAGGAACTACT
CHS-R	CAAATTCAACCCCAAGAACAAATGA
CHI-F	AGAAACCAGGAACCGAAAAATCA
CHI-R	CCTCTACTACGGCAACAAATGAC
F3′5′H-F	AACAACTTCTCCAACCGTCCAC
F3′5′H-R	CAAGGCTTTCCCCCCTAACA
18S ribosomal-F	AACCATAAACGATNCCGACCAG
18S ribosomal-R	NCTTGCGACCATACTCCC

Supplenmentary Table2. The 52 kinds of volatile components were detected in different days of *B. acuminata*

No.	Compouds	Relative contents (%)		
		1d	3d	5d
1	Linalool	37.59±8.39	31.27±2.12	19.24±4.12
2	2-Hexenal, (E)-	44.92±5.11	20.98±1.29	--
3	trans-Linalool oxide (furanoid)	5.91±1.47	11.61±1.58	8.27±1.56
4	(E)-4,8-Dimethylnona-1,3,7-triene	9.62±5.28	9.81±1.07	10.11±6.31
5	2-Furanmethanol, 5-ethenyltetrahydro-à,à,5-trimethyl-, cis-	3.47±0.42	9.34±1.60	6.07±1.37
6	1-Hexanol	5.22±0.83	5.13±1.25	12.53±5.98
7	Benzeneacetaldehyde	0.67±0.20	4.15±1.70	11.40±0.85
8	Benzaldehyde	0.48±0.10	1.90±0.83	5.14±0.78
9	(3R,6S)-2,2,6-Trimethyl-6-vinyltetrahydro-2H-pyran-3-ol	0.64±0.03	1.39±0.56	1.09±0.13
10	á-Ocimene	1.39±1.20	1.15±0.79	1.36±1.36
11	2-Butenoic acid, 2-methyl-, 3-methylbutyl ester, (E)-	--	1.00±0.06	0.48±0.16
12	Heptanal	1.46±0.24	0.95±0.23	0.65±0.01
13	2-Hexenal	0.74±0.44	0.45±0.13	16.78±1.43
14	2-Hexen-1-ol, (E)-	0.18±0.05	0.50±0.48	0.41±0.24
15	Phenylethyl Alcohol	--	0.21±0.04	1.04 ±0.17
16	Nerolidol	--	0.38±0.08	0.66±0.17
17	(3E,7E)-4,8,12-Trimethyltrideca-1,3,7,11-tetraene	--	0.59±0.21	--
18	Cinnamaldehyde, (E)-		0.31±0.14	0.31±0.04
19	Benzyl alcohol	0.10±0.02	0.24±0.01	0.48±0.03
20	2-Octenal, (E)-		0.23±0.07	0.44±0.07
21	2,4-Nonadienal, (E,E)-	0.20±0.04	0.21±0.03	0.35 ±0.07
22	Hexanal	0.37±0.12	0.20±0.11	0.07±0.04
23	2-Heptenal, (Z)-	0.36±0.08	0.19±0.01	0.24±0.01
24	Oxygen	0.63±0.43	0.17±0.05	0.15±0.01
25	1-Octanol	0.07± 0.04	0.16±0.01	0.22±0.04
26	2,4-Hexadienal, (E,E)-	0.24±0.09	0.13±0.03	0.08±0.01
27	(1S)-2,6,6-Trimethylbicyclo[3.1.1]hept-2-ene	0.08±0.03	0.12±0.04	0.11±0.05
28	Hexyl tiglate	0.15±0.03	0.10±0.02	0.06±0.02
29	Cyclopropane, propyl-	--	0.10±0.03	--
30	2-Hexenoic acid, methyl ester	--	0.09±0.02	0.05±0.01
31	Methional	0.10±0.00	0.09±0.01	0.11±0.01
32	(Z)-(Z)-Hex-3-en-1-yl 2-methylbut-2-enoate	0.09±0.03	0.09±0.04	--
33	Methyl tiglate	--	0.08±0.02	0.05±0.02
34	2,4-Heptadienal, (E,E)-	0.13±0.01	0.07±0.01	0.08±0.01
35	1-Heptanol	0.08±0.02	0.07±0.02	0.10±0.03
36	2,6-Octadienal, 3,7-dimethyl-, (E)-	--	0.07±0.03	--
37	Benzoic acid, hydrazide	--	0.07±0.02	--
38	Acetic acid, hexyl ester	--	0.06±0.02	0.08± 0.03
39	Naphthalene,	--	0.05±0.01	0.23± 0.05

	1,2,3,5,6,8a-hexahydro-4,7-dimethyl-1-(1-methy lethyl)-, (1S-cis)-			
40	Octanal	0.08± 0.01	0.05±0.03	--
41	Decanal		0.03±0.00	0.02± 0.01
42	1-Octen-3-one	0.05± 0.04	0.02±0.00	0.04± 0.01
43	1,3,5,7-Cyclooctatetraene	--	0.01±0.00	--
44	2,4,6-Trimethyl-1-nonene	--	0.01±0.00	--
45	4-Methyl-2,4-bis(p-hydroxyphenyl)pent-1-ene, 2TMS derivative	--	0.00±0.00	--
46	2-Nonenal, (E)-	0.05±0.01	0.05±0.00	0.06± 0.00
47	2,3-Hexanedione	0.88 ±0.08	--	--
48	1-Octen-3-ol	0.12±0.03	--	--
49	Oxime-, methoxy-phenyl-_	0.07±0.01	--	--
50	1,3-Dioxolane-2-methanol	--	--	0.04±0.01
51	1-Butanol, 3-methyl-, benzoate	--	--	0.03±0.01
52	1-Pentanol, 4-methyl-	--	--	0.01 ±0.00
